# Advancements in microenvironment-based therapies: transforming the landscape of multiple myeloma treatment

**DOI:** 10.3389/fonc.2024.1413494

**Published:** 2024-07-17

**Authors:** Ke Lu, Wen Wang, Yuntong Liu, Chao Xie, Jiye Liu, Lijie Xing

**Affiliations:** ^1^ Department of Lymphoma, Shandong Cancer Hospital and Institute, Shandong First Medical University and Shandong Academy of Medical Sciences, Jinan, Shandong, China; ^2^ Jerome Lipper Multiple Myeloma Center, LeBow Institute for Myeloma Therapeutics, Dana-Farber Cancer Institute, Harvard Medical School, Boston, MA, United States; ^3^ Department of Respiratory, Shandong Cancer Hospital and Institute, Shandong First Medical University and Shandong Academy of Medical Sciences, Jinan, Shandong, China; ^4^ Key Laboratory of Biopharmaceuticals, Postdoctoral Scientific Research Workstation, Shandong Academy of Pharmaceutical Science, Jinan, Shandong, China

**Keywords:** bone marrow microenvironment, cellular compartments, noncellular compartments, multiple myeloma, treatment

## Abstract

Multiple myeloma (MM) is the most prevalent malignant monoclonal disease of plasma cells. There is mounting evidence that interactions with the bone marrow (BM) niche are essential for the differentiation, proliferation, survival, migration, and treatment resistance of myeloma cells. For this reason, gaining a deeper comprehension of how BM microenvironment compartments interact with myeloma cells may inspire new therapeutic ideas that enhance patient outcomes. This review will concentrate on the most recent findings regarding the mechanisms of interaction between microenvironment and MM and highlight research on treatment targeting the BM niche.

## 1 Introduction

Multiple myeloma (MM) is characterized by clonal expansion of malignant plasma cells within the bone marrow (BM), leading to organ failure at the time of diagnosis, which includes calcium elevation, renal dysfunction, anemia, and/or bone disease (CRAB) (1). Remarkable discoveries in the biology of MM have transformed the treatment paradigm and extended median patient survival over the past two decades. Nonetheless, MM remains mostly incurable due to genetic complexity and instability, as well as the permissive, tumor-promoting BM microenvironment. Thus, new therapeutics targeting both myeloma cells and immunosuppressive microenvironment are urgently needed.

**Figure 1:**
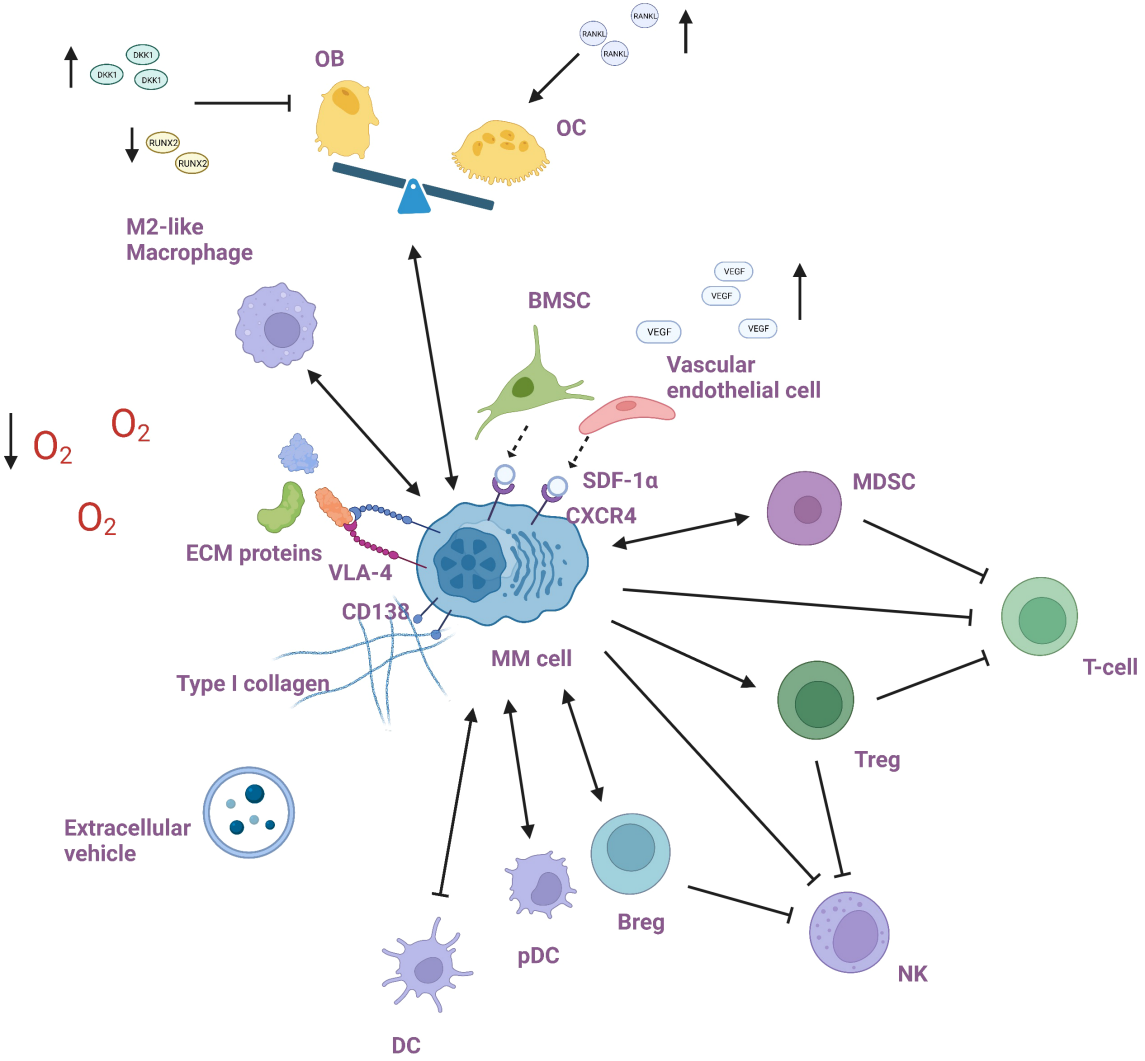
Bone marrow microenvironment of multiple myeloma.

The BM microenvironment is a heterogeneous system comprising a cellular compartment (e.g., immune cells (including myeloid-derived suppressor cells, dendritic cells, macrophages, T-cells, natural killer cells, regulatory B cells), osteoblasts, osteoclasts, endothelial cells, and stromal cells) and a non-cellular compartment [e.g., extracellular matrix (ECM), extracellular vehicles (EVs), oxygen concentration, and the liquid milieu (cytokines, growth factors, and chemokines)] (**Figure 1**). The interaction between myeloma cells and the BM milieu encourages tumor immune escape and promotes the former’s proliferation, survival, dissemination, and drug tolerance via a variety of mechanisms (2–4). In this review, we describe the mechanisms by which each BM milieu member promotes the development of MM cells and outline potential therapies to target them.

## 2 Cellular compartments of the BM microenvironment

### 2.1 Myeloid-derived suppressor cells

MDSCs are a diverse population of immature myeloid cells (IMCs) that, under normal conditions, can differentiate into granulocytes, macrophages, or dendritic cells under steady-state settings (5). However, in pathological conditions typical of malignancies, the differentiation of IMCs was reported to be inhibited, resulting in the accumulation of MDSCs. Current studies identify two main subtypes of MDSCs: monocytic myeloid-derived suppressor cells (M-MDSCs), which are phenotypically and morphologically similar to monocytes, and granulocytic/polymorphonuclear myeloid-derived suppressor cells (PMN-MDSCs), which are phenotypically and morphologically similar to neutrophils. A significant presence of MDSCs is found in the multiple myeloma tumor microenvironment, where they play a crucial role in immune escape and disease progression (6, 7). It has become clear that there is a bidirectional interaction between MM cells, MDSCs, and immune effector cells. It has been demonstrated that M-MDSC plays a more predominant immunosuppressive role in the multiple myeloma tumor microenvironment. As a precursor of osteoclasts, M-MDSC contribute to bone destruction, leading to severe bone pain or pathological fractures in some MM patients. Conversely, PMN-MDSCs are more involved in neovascularization mechanisms within the bone marrow microenvironment (6, 8). MDSCs exert immunosuppressive effects through multiple mechanisms: they produce and release inhibitory cytokines and soluble factors such as IL-10 and TGF-β, and induce regulatory T cells (Tregs) (9, 10); they generate reactive oxygen species (ROS), which disrupt the ability of CD8+ T cells to bind to peptide-major histocompatibility complexes (11). MDSCs also reduce tryptophan levels in the tumor microenvironment due to the expression of Arg1 and Indoleamine 2,3-Dioxygenase (IDO) enzyme activity, which diminishes TCR formation, inhibits T-cell proliferation and induces T-cell cycle arrest by depletion of L-arginine (12, 13). Additionally, MDSCs can increase the levels of anti-apoptotic proteins in MM cells by inducing AMPK phosphorylation, thereby enhancing the proliferation of MM cells (14). They also endow stem-like qualities to myeloma cells, promoting epithelial-mesenchymal transition (EMT) (15). Conversely, malignant plasma cells stimulate the growth of MDSCs (16).

### 2.2 Dendritic cells

DCs are expert antigen-presenting cells (APCs) that deliver antigens to T cells and, depending on their functional condition, either induce immunity or tolerance. Two main subgroups of DCs can be distinguished by their origin, phenotype, and function: myeloid DCs (mDCs) and plasmacytoid DCs (pDCs) (17). In the literature, there is no universal consensus on the phenotype, function, and frequency of DC population in MM patients compared to healthy individuals. Nonetheless, considerable research has shown that DCs in MM patients’ BM are dysfunctional, resulting in diminished anti-tumor immune responses and myeloma escape (18–20). It has been discovered that interactions between DCs and MM cells can confer proliferation, survival, and medication resistance against tumor cells through receptor activator of nuclear factor-κB (RANK)/RANK ligand (RANKL) signaling and a proliferation-inducing ligand (APRIL)-mediated interactions (21), as well as CD28 and CD80/CD86 crosslinking (22, 23). In addition, pDC-MM interaction triggers the secretion of cytokines and chemokines, which not only helps MM cells grow, survive, and develop treatment resistance, but also extends the survival of pDCs (24). More recently, Ray et al. used next-generation sequencing (NGS) to examine genomic alterations in MM cells induced by co-culture with pDCs. They discovered that co-culturing pDCs with myeloma cells increases CD73, CD274, HDAC6, TLR7/9, or IL3Ra/CD123 gene expression, while reducing the expression of ADAM33, BAD, BAK1, and CASP3 in tumor cells. These results make it possible to induce MM-specific CD8+ CTL activity by blocking CD73. Furthermore, combining an anti-CD73 antibody with a TLR7 agonist significantly increases the cytotoxic activity of MM-specific CD8+ CTL (25). Altogether, these findings highlight the importance of DCs in the etiopathogenesis of MM and present novel therapeutic targets to improve patient outcomes.

### 2.3 Macrophages

Macrophages are known as specialized phagocytic cells that are essential for both innate immune response and tissue repair. There are two main subsets of macrophages: M1 type macrophages (M1) and M2 type macrophages (M2). In the tumor setting, M1 typically acts as a potent anti-tumor effector and kills tumor cells by mediating direct cytotoxicity and antibody-dependent cell-mediated phagocytosis (ADCP). In contrast, M2 facilitates tumor growth, invasion, metastasis, and treatment resistance (26). Tumor-associated macrophages (TAMs) originate from circulating monocytes, which are recruited to the tumor site by cytokines released by tumor cells (27, 28). In comparison to Monoclonal Gammopathy Of Undetermined Significance (MGUS), MM exhibits higher expression of TAMs, suggesting their potential role in the transition from MGUS to MM (29, 30). Previous research indicates that during disease progression, the tumor microenvironment in MM undergoes macrophage reprogramming. Reprogrammed TAMs demonstrate a mixed phenotype, displaying characteristics of both M1 and M2 macrophages, with a predominant M2-like profile. These reprogrammed TAMs are associated with impaired phagocytic function (30, 31). Multiple myeloma cells recruit macrophages that support tumor growth and encourage their polarization toward an M2-like phenotype via several different mechanisms (29, 32–34). TAMs exert multifaceted effects  on MM, influencing proliferation, migration, angiogenesis, immunosuppression, and drug resistance (35). They promote MM cell proliferation by upregulating secretion of IL-6 and IL-10 (36–38), and enhance migration by inducing cytokine-mediated vascular leakage and downregulating CD138 and C-X-C motif chemokine receptor 4 (CXCR4), thus reducing cell adhesion (39–41). TAMs contribute to angiogenesis both directly and indirectly: they produce and release angiogenic factors such as vascular endothelial growth factor (VEGF) and matrix metalloproteinases (MMPs), and *in vitro*, they cooperate with MM cells to stimulate proliferation, migration, and tubule formation of Human Umbilical Vein Endothelial Cells (HUVECs). Additionally, TAMs exposed to VEGF and basic fibroblast growth factor can directly form capillary-like blood vessels by acquiring endothelial cell markers (42–44); Moreover, TAMs induce immunosuppression by downregulating IFN-γ, inhibiting MHC Class II molecule expression to limit effector T cell function, and modulating immunosuppression through the macrophage immune checkpoint CD47-SIRPα (29, 41, 45, 46); Studies also indicate that TAMs contribute to increased drug resistance in MM cells (47, 48). To sum up, MM cells can manipulate macrophages to facilitate tumor settlement and progression. Accordingly, anti-TAM therapeutic strategies are emerging as intriguing and promising approaches. Much evidence indicates that TAMs contribute to MM cell resistance to chemotherapeutic drugs. TAMs can mediate bortezomib resistance by secreting IL-1β, which increases the number of MM-tumor-initiating cells (49). Additionally, TAMs can express B-cell activating factor (BAFF), preventing bortezomib-induced apoptosis through the classical and alternative NF-κB pathways (50). Moreover, TAMs impact the efficacy of CAR-T therapy. A report showed that in B cell NHL, patients achieving complete responses with CAR-T therapy had decreased levels of TAMs, Treg cells, and MDSCs, whereas chemokines and MDSCs were overexpressed in patients achieving only partial remission (51).

### 2.4 T-cells

The progression of MM is associated with a deteriorating innate and adaptive immune system, particularly affecting the T-cell repertoire. CD8+ cytotoxic T lymphocytes (CTLs) mediated cytotoxicity plays a pivotal role in anti-tumor T-cell responses (52). However, T-cell dysfunction has been observed even in individuals at precursor stages of plasma cell dyscrasia, such as increased levels of T-cell exhaustion in monoclonal gammopathy of undetermined significance (MGUS) (53) and reduced expression of activation markers in smoldering multiple myeloma (SMM) (54). Notably, T-cell function progressively deteriorates throughout the disease course. In MM patients, especially those with relapsed/refractory MM (RRMM), co-inhibitory molecules including PD-1 and TIGIT are upregulated on activated T-cells, which protects myeloma cells from immune attack by directly interacting with their ligand expressed in myeloma cells (55–57). However, up to now, immune checkpoint inhibitors (ICIs) have failed to show promising clinical benefits in MM, possibly due to compromised antigen-specific T-cell function or loss of stem-like/tissue-resident memory T (TRM) cells (53, 58).

CD4+ T-cells can be categorized into several functionally distinct T-cell subsets: T helper 1 (TH1), TH2, TH17, TH22, T follicular helper (TFH), and regulatory T-cells (Tregs) (59). The specific immune imbalance of CD4+ T-cell subsets on MM remains unclear and controversial. Nevertheless, several research findings are more widely accepted. Firstly, TH1 cells collaborate with tumor-infiltrating, antigen-presenting macrophages to achieve anti-tumor responses (60). Then, MM cells induce the generation of Tregs via multiple mechanisms (61–65), contributing to immune dysfunction and negatively influencing clinical outcomes (66). Lastly, in MM patients, there is a significant increase in TH17 cells and related cytokines, such as IL-17, which promotes MM cell proliferation while inhibiting immunological responses (67–70). Strikingly, TH2 cells, which are generally considered to have a pro-tumorigenic role, have been shown to eradicate myeloma by triggering an *in situ* inflammatory immune response (71).

In recent years, unconventional T cells have garnered increased attention for their pivotal role in hematological tumors. Among these, γδ T cells, MAIT cells, and iNKT cells (invariant natural killer T cells) exhibit both innate and adaptive immune characteristics, primarily characterized by their rapid recognition of unconventional peptide antigens. Stimulated γδ T cells demonstrate potent cytotoxic effects on MM cells *in vitro (* 72), with Vδ1 cells being activated by various receptors, including T-cell receptors and molecules such as NKG2D, CD3, CD2, DNAX accessory molecule-1, and intracellular adhesion molecule-1 (ICAM-1). This activation confers cytotoxic capabilities against MM cells, suggesting a potential therapeutic strategy for MM (73). Additionally, studies have indicated a reduction in the percentage of MAIT cells in MM patients compared to healthy individuals, accompanied by decreased production of IFN-γ and reduced CD27 expression in MAIT cells at disease onset, indicative of MAIT cell depletion (74).

### 2.5 Natural killer cells

Natural killer (NK) cells are vital to the innate immune response due to their direct cytotoxic activity and antibody-dependent cellular cytotoxicity (ADCC). Previous studies have shown that NK cell reactivity is mediated by the expression of a wide variety of inhibitory receptors characterized by CD94-NKG2A heterodimeric receptors, killer cell Ig-like receptors (KIRs), as well as activating receptors such as CD16, NKG2D, DNAM-1, activating KIR, and the natural cytotoxicity receptor (NCR) family (75). Malignant plasma cells can evade NK cell-mediated killing by downregulation or blocking activating receptors and activating Tregs or regulatory B cells (Bregs) (76–79).

### 2.6 Regulatory B cells

The potential effects of B-cell subsets on the BM milieu in MM are not well characterized. However, emerging research suggests a small immunosuppressive B-cell subset known as Bregs has the ability to mediate evasion of myeloma plasma cells from the immune system. Malignant plasma cells in the bone marrow support the survival of Bregs by preventing their apoptosis and activating the APRIL/TACI axis (63, 79). Consequently, Bregs promote an immune suppressive microenvironment through the production of IL10 and alternative mechanism including the interference with NK cell-mediated ADCC against MM cells. Thus, Bregs are a promising new therapeutic target in MM.

### 2.7 Osteoblasts (OBs) and osteoclasts (OCs)

In a steady state, the differentiation and activity of OCs and OBs maintain a balance between bone resorption and formation, ensuring bone homeostasis and integrity. In MM environment, the OC-OB axis is disrupted leading to increased bone resorption and impaired bone formation, ultimately resulting in osteolytic bone disease. In MM, tumor PCs produce both activators of OCs and inhibitors of OBs (80): MM cells increase the differentiation and activity of OCs by dramatically increasing the release of osteoclastogenic factors (81–87) and favoring the recruitment of OCs precursors (88–90), conversely, malignant plasma cells can elevate Dickkopf-1 (DKK1) levels (91) and decrease the essential OB transcription factor RUNX2 activity (92) leading to the suppression of osteoblast activity and osteoblast differentiation. OBs play a crucial role in bone formation by producing bone morphogenetic protein (BMP), which binds to receptor and activates downstream osteoblast-specific transcription factor RUNX2,OSTERIX, etc (93). Additionally, Wnt in OBs binds to LRP-5/6, activates β-catenin and promotes the differentiation of bone marrow mesenchymal stem cells to osteoblasts while inhibiting their apoptosis (94). Sclerostin (SOST), primarily secreted by osteoblasts, serves as a significant negative regulator of bone formation. Elevated levels of SOST expression hinder the Wnt and BMP signaling pathways, thereby impeding osteoblast differentiation and proliferation and ultimately inhibiting bone formation (95). Importantly, recent studies reveal that the function of OCs and OBs not only contributes to bone-remolding, but also involves maintaining an immunosuppressive myeloma environment. OCs shield myeloma cells from T-cell cytolytic function via high expression of checkpoint molecules including PD-L1, IDO HVEM, CD200, and Galectin-9 (96). Myeloma-osteoclast interactions upregulate Chondroitin synthase 1 (CHSY1), triggering Notch signaling and boosting tumor cell proliferation and bone resorption (97). Additionally, through the modification of the endosteal niche, OCs are able to control the resurgence of dormant myeloma cells (98, 99). Lastly, OCs encourage angiogenesis, which is necessary for the survival and multiplication of MM cells (100, 101). As for OBs, Khoo WH et al. have found that the interactions between MM and osteoblasts may also play a role in maintaining tumor cell dormancy (102). Taken together, the above findings underline the therapeutic value of targeting OCs to modulate BM microenvironment, improve anti-tumoral immune responses, and improve the bone phenotype.

### 2.8 Bone marrow stromal cells and vascular endothelial cells

Tom Cupedo et al. conducted a comprehensive mapping of the myeloma inflammatory stromal microenvironment using single-cell transcriptome sequencing. They identified myeloma-specific inflammatory stromal cells that spatially colocalize with tumor and immune cells, are induced by inflammatory factors, and provide essential survival factors to plasma cells. The MM microenvironment was characterized by the expansion of IFN-responsive T cells, CD8^+^ Tscm cells and GZMK^+^CX3CR1^-^CD56^bright^ NK cells, while the distribution of other T cells, B cells and mononuclear myeloid cells remain unaltered (103). Additionally, Tom Cupedo and colleagues reported that neutrophils in the bone marrow of MM patients are activated to promote the transcription of IL-1β and myeloma cell survival factor TNFSF13B (BAFF). These neutrophils establish a positive feedback loop with inflammatory stromal cells, thereby perpetuating a tumor-supportive inflammatory environment after treatment (104). BMSCs and vascular endothelial cells can produce a chemoattractant called stromal cell-derived factor 1 alpha (SDF-1a). The binding of SDF-1a to its receptor CXCR4 is involved in the mobilization and homing of hematopoietic stem and progenitor cells (105). In MM, myeloma cells utilize CXCR4 to interact with SDF-1a, resulting in the adhesion of myeloma cells to BMSCs and endothelial cells. This interaction leads to the overexpression of factors such as vascular endothelial growth factor (VEGF), hepatocyte growth factor (HGF), IL-6, IL-3, and tumor necrosis factor alpha (TNFα) which contributes to osteolysis, angiogenesis, as well as MM cell survival and proliferation (106). The binding of malignant plasma cells to BMSCs also triggers the activation of various adhesion molecules such as very-late-antigen-4 (VLA-4) and CD44, which mediates cell adhesion-mediated drug resistance (CAM-DR) (107). Recent studies revealed that CXCR4 could enhance the acquisition of epithelial-to-mesenchymal transition (EMT-like) phenotype in MM cells, promoting extramedullary disease (EMD) development both *in vivo* and *in vitro (* 40, 108).

## 3 Non-cellular compartments of the BM microenvironment

The BM extracellular matrix (ECM) consists of proteins such as fibronectin, collagen, osteopontin, hyaluronan, and laminin, serving as a scaffold for tumor cells to cling to and interact with its components or other cells. MM cells directly interact with the ECM by binding integrins like VLA-4 and integrin b7 (ITGB7) to ECM proteins. This interaction is required for MM cell survival and contributes to CAM-DR (109–112). Besides, CD138 expressed on MM cells binds to type I collagen and promotes matrix metalloproteinase 1 (MMP1) production, thereby stimulating tumor invasion, bone resorption, and angiogenesis (113, 114).

Another important component of BM microenvironment is extracellular vehicles (EVs). EVs are a diverse class of membranous structures released by all kind types of cells and act as important intermediaries between myeloma cells and the surrounding milieu or remote premetastatic niche (115). Recent researches have uncovered the crucial role of EVs in tumor progression through various mechanisms, including but not limited to angiogenesis moderation (116–118), participation in the formation of bone lesions (119–122), mesenchymal cell education (123–126), and immune modulation (127–130).

In addition, it has been demonstrated that the BM of MM mouse models and MM patients is hypoxic compared to healthy controls (131, 132). The decrease in oxygen concentration enhances the acquisition of stem cell-like or EMT-like phenotype in MM cells, thus enhancing their dissemination properties (133, 134). Also, hypoxia contributes to the progression of osteolytic bone disease as well as angiogenesis (135, 136). Targeting the hypoxia microenvironment should be considered as a novel anti-MM treatment strategy and has the potential to synergize with other anti-MM therapies (137).

## 4 Treatment targeting MM microenvironment components

**Figure 2:**
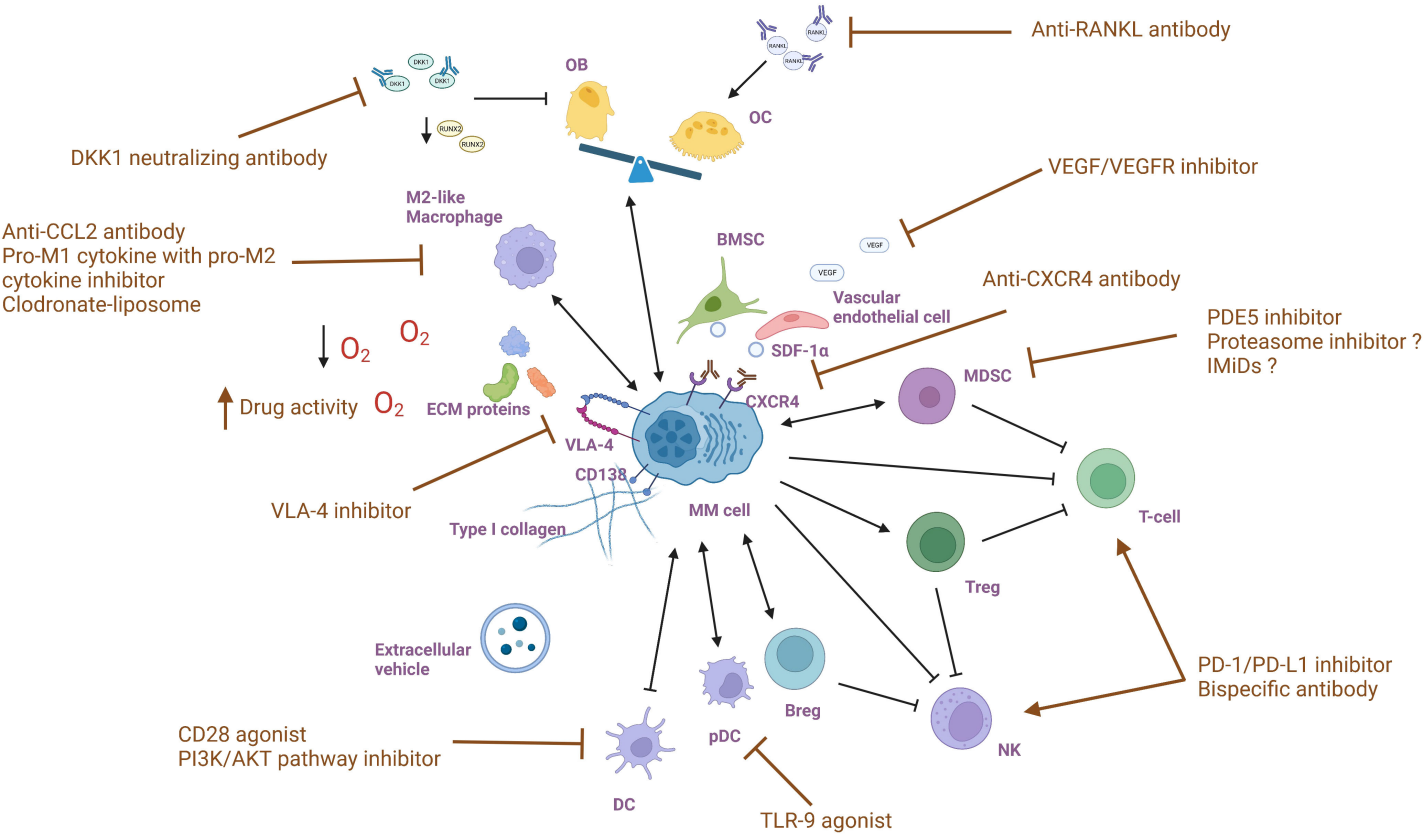
Targeting bone marrow microenvironment components in multiple myeloma.

MM treatments targeting the BM microenvironment aim to disrupt the complex interactions between MM cells and the surrounding environment and restore certain cellular functions, altering the supportive environment for MM cells. By understanding the molecular and cellular changes that occur in the BM microenvironment in response to MM, the drugs are selected or designed to target specific components and pathways that are affected by MM (**Figure 2**). Multiple drugs have shown efficacy in interfering with MM cell growth, survival, and drug resistance (**Table 1**).

**Table 1:** Strategies targeting BM microenvironment of MM.

Targeting component	Approach (Agent)	Stage	Findings
MDSCs	Proteasome inhibitor (bortezomib), IMiDs (lenalidomide)	Preclinical in cells	Contradicting results
	PDE5 inhibitor (tadalafil)	Preclinical in myeloma mouse models; Case report	Decreased suppression by tumor cells; Generated durable anti-tumor immune and clinical response in one RRMM patient
DCs	TLR-9 agonist	Preclinical in cells	Restored pDC immune; Inhibited MM proliferation
	CD28 agonist (abatacept)	Phase 2 clinical trial in MM (NCT03457142)	Combined with ixazomib and dexamethasone; ORR 33.33%, limited AEs
	PI3K/AKT pathway inhibition	Proposed	
Macrophages	Anti-CCL2 mAb (carlumab)	Phase 1b clinical trial in solid tumors (NCT01204996)	Combined with one of four chemotherapy regimens; well-tolerated, no significant tumor response; not tested in MM
	Reprogram toward an M1 profile	Preclinical in MM xenograft models	Combining pro-M1 cytokine with pro-M2 cytokine inhibitor produced anti-tumor response
	Deplete mature macrophage (clodronate-liposome)	Preclinical in myeloma mouse model	Depleted bone marrow-resident macrophages and reduced myeloma tumor burden
T-cells and natural killer cells	Reinvigorate immune cell function (PD-1/PD-L1 inhibitor)	Phase 1b single-drug trial (e.g., KEYNOTE-013, NCT01592370); Phase 3 combination therapy trials (e.g., KEYNOTE-183, KEYNOTE-185)	No efficacy in MM patients as single agent; combination therapy with IMiDs in RRMM and newly diagnosed MM showed excessive mortality; discontinued by FDA
	Enhance interaction between immune cells and MM cells (bispecific antibody)	Preclinical in cells	Bispecific antibody promoted the formation of immune synapses and activated cytolytic immune cells
	CAR-T	phase 1 and phase 3 studies in RRMM (NCT02658929) (NCT03651128)	Improving objective remission rates and prolonging progression-free survival in RRMM patients
Osteoclasts	Target RANKL (denosumab)	Phase 3 clinical trial (NCT01345019)	Similar efficacy in preventing skeletal-related events in newly diagnosed MM as compared to zoledronic acid; better progression-free survival endpoint
Osteoblasts	Target DKK1 (BHQ880)	Phase 1b clinical trial (NCT00741377)	Combination with zoledronic acid was well tolerated in RRMM; relative single agent clinical activity was not assessed
BMSCs and vascular endothelial cells	Disrupt interaction between MM cells and BM milieu (ulocuplumab)	Phase 1b/2 clinical trial (NCT01359657)	Ulocuplumab alone was safe with acceptable AEs in RRMM; combination with lenalidomide and dexamethasone resulted in 55.2% ORR
	VEGF or VEGF receptor inhibition (e.g., bevacizumab, pazopanib)	Phase 2 clinical trials (e.g., GW786034, AMBER)	Neither single agent nor combination therapy with IMiDs or bortezomib showed meaningful clinical activity
Non-cellular	Disrupt interaction of MM cells with BM ECM (natalizumab)	Phase 1/2 clinical trial (NCT00675428)	Terminated because of low patient enrollment
	Improve drug activity in hypoxic condition (evofosfamide)	Phase 1/2 clinical trial (NCT01522872)	Single agent or in combination with bortezomib had good tolerability and clinical activity in RRMM

### 4.1 MDSCs

Given that currently available medicines like the proteasome inhibitor and the immunomodulatory drug target both myeloma cells and the MM milieu, the influence of these treatments on MDSCs has been investigated. Görgün and colleagues showed neither bortezomib nor lenalidomide eliminated the amount or suppressive activity of MDSCs *in vitro (* 138). However, in contrast, another study from Wang et al. discovered a decrease in MDSCs after proteasome inhibitor therapy (139). These contradicting results indicate that the impact of currently accessible treatments on MM MDSCs is still debatable and requires further research. Phosphodiesterase-5 (PDE5) inhibition in preclinical studies has demonstrated down-regulation of expression of arginase 1 and nitric oxide synthase-2 in murine tumor models, thereby decreasing the suppressive machinery of MDSCs recruited by tumor cells (140). Noonan et al. reported a case where the addition of tadalafil, a PDE5 inhibitor, restored the responsiveness to lenalidomide-based therapy in one patient who was previously refractory to lenalidomide (141). These data indicate that strategies targeting the function and amount of MDSCs with PDE5 inhibitors may provide a unique strategy that can synergize with tumor-directed therapies to generate a significant and long-lasting anti-myeloma immune and clinical response.

### 4.2 DCs

Currently explored MM therapeutic strategies involve pDC immune function restoration and DCs-mediated myeloma proliferation inhibition. Preclinical data have demonstrated that Toll-like receptor 9 (TLR-9) agonists can restore pDC immune function and simultaneously abrogate pDC-induced MM cell growth (142). Unfortunately, there is no clinical trial of TLR-9 agonist in MM patients so far. *In vitro* studies have demonstrated that blocking of RANKL: APRIL and CD28: CD80/CD86 pathways can suppress myeloma growth mediated by DCs and resensitize tumor plasma cells to lysis by cytotoxic T cells (21, 22). Currently, a synthetic antagonist of CD28, abatacept is being assessed in conjunction with ixazomib and dexamethasone in a phase 2 clinical trial (NCT03457142) for participants with chemotherapy-resistant multiple myeloma. Another therapeutic target is suggested by Megan et al. as they reported that CD28 pro-survival signaling relies on the phosphoinositide 3-kinase (PI3K)/protein kinase B (AKT) pathway (143). This pathway is aberrantly activated through numerous mechanisms in most MM patients and is essential for MM survival and chemotherapy resistance (23). As such, PI3K/AKT pathway inhibitors may block CD28 signaling and resensitize MM cells to chemotherapies.

### 4.3 Macrophages

Existing therapeutic approaches targeting macrophages mainly include blocking their recruitment to the tumor microenvironment, reprogramming TAMs, inhibiting the CD47/SIRPα checkpoint, and reversing drug resistance (35). Clodronate Liposome directly deplete BM-resident macrophages and disrupts MM cell homing (144). Reducing monocyte/macrophage recruitment by modulating growth factors, chemokines and cytokines in tumor and stromal cells also diminishes the supportive role of TAMs. The CXCL12-CXCR4 signaling pathway is crucial for macrophage recruitment (145). High expression of the chemokine CXCL12 in MM cells promotes monocyte recruitment and differentiation into an M2 phenotype, which enhances angiogenesis and immunosuppression (29). CXCR4 antibody significantly reduces monocyte recruitment. MM secretes CCL2, which induces the expression of monocyte chemotactic protein-1-induced protein 1(MCPIP1) in macrophages via the JAK2-STAT3 pathway, promoting macrophage homing, proliferation, and M2-like phenotypic polarization (33, 34). Consequently, CCR2 monoclonal antibodies or inhibitors can disrupt macrophage recruitment in MM (146, 147).

Reprogramming TAMs involves decreasing the M2 immunosuppressive phenotype and increasing the M1 phenotype. Gutiérrez-González et al. suggest that the combination of pro-M1 cytokine granulocyte-macrophage CSF (GM-CSF) and pro-M2 cytokine macrophage migration inhibitory factor inhibitor has significant anti-tumor effects (148). Preclinical studies have shown that the JAK1/2 inhibitor Ruxolitinib reduces the M2 polarized phenotype and increases the M1 polarized phenotype by downregulating Tribbles Homolog 1 protein kinase expression (149). Both CD40 agonists and blockers of IL-10R have been shown to reprogram TAMs (150, 151).

Regarding immune checkpoints, numerous CD47-targeted drugs, including anti-CD47 monoclonal antibodies and SIPRα fusion proteins, are under clinical investigation (152). AO-176 is a humanized IgG2 anti-cd47 monoclonal antibody, is being evaluated in a phase 1/2 clinical study to assess its efficacy as a monotherapy and in combination with bortezomib/dexamethasone for the treatment of MM (NCT04445701).

### 4.4 T-cells and NK cells

Reinvigorating T-cells and NK cells is considered a compelling and promising therapeutic option in MM. As previously discussed, the increased expression of immune checkpoints in T- and NK cells in MM results in impaired cytolytic immune cell function and the establishment of a tumor-promoting and immune-suppressive microenvironment. Inhibition of PD-1/PD-L1 has shown potential as a treatment for myeloma based on preclinical evidence (153, 154). However, early-phase clinical trials targeting PD-1/PD-L1 showed no efficacy when using a single-drug approach (155, 156). As a result, subsequent studies have focused on investigating the blockade of the PD-1 axis as part of a combination treatment approach with immunomodulatory drugs (IMiDs). One of the IMiDs, lenalidomide, has been proven to enhance checkpoint blockade-induced MM cytotoxicity in a preclinical study (154). Encouragingly, early clinical studies have shown acceptable safety and durable responses of this treatment (157). Unfortunately, two phase 3 trials (KEYNOTE-183 and KEYNOTE-185) evaluating the efficacy and safety of combining PD-1 inhibitor pembrolizumab with IMiDs for the treatment of RRMM and newly diagnosed MM revealed excessive mortality (158, 159). These adverse outcomes led to the discontinuation of these two clinical trials by the Food and Drug Administration (FDA), along with several other similar studies. Based on these findings, the key points in future studies should be to identify patients who would benefit most from checkpoint targeting, determine appropriate drug combinations, and effectively manage adverse events. Furthermore, ongoing research is exploring the benefit-risk profile of the other immune checkpoint or agonist proteins, such as T-cell immunoreceptor with Ig and ITIM domains (TIGIT), lymphocyte activation gene-3 (LAG3), OX40, and immunoglobulin-like receptors (KIRs), as prospective therapeutic targets for MM, either alone or in combination with MM targeted and immunotherapies (160, 161).

An alternative approach is to enhance the interaction between immune cells and MM cells. The vast majority of cytolytic immune cells, such as CD8+ T cells, NK cells, and NK T cells, express an activation receptor called NKG2D. Meanwhile, CS1 (SLAMF7), a surface lymphocytic activation molecule, is highly expressed on MM cells compared to NK cells and a subset of activated T-cells. Building upon this knowledge, Wing Keung and colleagues engineered a bispecific antibody (biAb) to bring together immune cells and MM cells by combining an anti-CS1 single-chain variable fragment (scFv) with an anti-NKG2D scFv (CS1-NKG2D biAb). They demonstrated that the CS1-NKG2D biAb effectively engaged human MM cell lines and NKG2D+ cytolytic innate and antigen-specific effector cells. This interaction facilitated the formation of immune synapses and activated these immune cells against MM (162). The compelling results from this study warrant further research into the effects and potential benefits of CS1-NKG2D biAb in the context of MM treatment.

Currently, novel T-cell-based immunotherapies such as chimeric antigen receptor (CAR-T) therapy and bispecific T cell engagers (BiTE) have significantly improved the treatment of MM. CAR-T therapy involves genetically modifying a patient’s T cells *in vitro* to express receptors that target specific antigens on tumor cells, enabling these T cells to effectively eliminate the tumor cells upon transfusion back into the patient. The primary targets for CAR-T in MM include B cell maturation antigen (BCMA), CD138, CD19, and CD38. BCMA is exclusively expressed on the surface of mature B cells, and its overexpression and activation are associated with MM in preclinical models and clinical studies, underscoring its potential as a therapeutic target (163). bb2121(ide-cel), a CAR-T therapy targeting BCMA, has demonstrated promising efficacy in phase I and phase III studies for patients with relapsed or refractory multiple myeloma (NCT02658929) (NCT03651128) (164, 165).

CD38, a transmembrane glycoprotein involved in calcium ion regulation, signal transduction and cell adhesion is highly expressed in B precursor cells, plasma cells, natural killer cells and bone marrow precursor cells. CAR-T therapies co-targeting CD38 and other tumor surface antigens, such as BCMA, have also been employed to treat recurrent MM.

The inflammatory response induced by CAR-T cells can enhance the recognition of neoantigens by the host immune system and trigger the anti-tumor response of the natural immune system. It promoted the recognition of other tumor antigens by unconventional T cells through TCR and helped to target tumor cells that were negative for CAR T cell antigens (166).

BiTE antibody molecules consist of single-chain fragment variable (scFvs) from two monoclonal antibodies that recognize antigens on the surface of target cells and CD3 molecules on the surface of T cells. By binding to both the target cell surface antigen and T cell CD3, BiTEs can activate the proliferation of polyclonal cytotoxic T cells, thereby exerting cytotoxic effects and killing target cells (163).

BiTEs targeting BCMA, GPRC5D (G protein-coupled receptor family C group 5 member D), and FcRH5 (Fc receptor-homologue 5) have shown good efficacy and manageable safety profiles in patients with relapsed/refractory multiple myeloma (RRMM) (167). Multiple clinical studies suggest that bispecific antibodies (BsAbs) provide significant survival benefits for RRMM patients. Currently, there are three BiTEs approved by the FDA for the treatment of multiple myeloma, targeting BCMA and GPRC5D.

Teclistamab, a BCMA × CD3 bispecific antibody, was the first approved for the treatment of RRMM. In the phase 1/2 MajesTEC-1 study, teclistamab demonstrated an overall response rate (ORR) of 63.0% in 165 heavily pretreated patients with RRMM (168, 169). Elranatamab, another BCMA × CD3 bispecific antibody, is used for the treatment of multiple myeloma. The approval of this drug is based on several pivotal clinical trials where elranatamab demonstrated high rates of deep and durable responses, including in patients achieving ≥CR. It also exhibited manageable safety (170, 171).

GPRC5D is a G protein-coupled orphan receptor with high expression on malignant plasma cells and low levels on B cells and bone marrow precursor cells, making it a promising target for RRMM patients. Talquetamab, a first-in-class GPRC5D × CD3 bispecific antibody, showed promising results in the MonumenTAL-1 study (NCT03399799) and (NCT04634552). With a median follow-up of 14.9 months, 8.6 months, and 11.8 months in cohorts receiving 0.4 mg/kg QW, 0.8 mg/kg Q2W, and prior T-cell redirected therapies, talquetamab achieved ORRs of 74%, 73%, and 63%, respectively. Cytokine release syndrome (CRS) and immune effector cell-associated neurotoxicity syndrome (ICANS) were observed in 79%, 75%, 77% and 11%, 11%, 3% of patients, respectively. The incidence of grade 3/4 infections was 22%, 16%, and 26%, respectively (172).

FcRH5 is expressed on almost all myeloma cells, with significant higher expression than normal B cells. The FcRH5-targeting BiTEs, Cevostamab (BFCR4350A, RG6160), demonstrated promising results in the GO39775 trial (NCT03275103), achieving an ORR of approximately 52% in RRMM patients, with only one patient (2%) experiencing grade 3 cytokine release syndrome (173).

Tri-specific antibodies, which can simultaneously target two tumor antigens, such as BCMA+GPRC5D/CD3, are under clinical investigation.

However, antibody-based drugs face challenges, particularly related to infection risks, necessitating careful management to prevent and treat infections like Pneumocystis pneumonia and viral infections. Immune-related adverse reactions include cytokine storms and immune-mediated lung injury. With GPRC5D bispecific antibodies, there are concerns about potential central nervous system damage and keratin-related changes (skin and nail alterations, taste loss, dry mouth, and swallowing difficulties). Patients still face significant hurdles and require additional treatment post-relapse (174, 175).

### 4.5 OCs and OBs

As for OCs, the current focus of MM treatment is to target the protein RANKL, which is overproduced by myeloma cells, bone marrow stromal cells, and osteocytes in MM. This excessive RANKL production leads to increased osteoclast activity which in turn recruits myeloma cells and promotes their proliferation, survival, and resistance to apoptosis (176–179). Denosumab, a human monoclonal antibody with high affinity and specificity for RANKL, has been studied as a potential treatment option. A phase 3 clinical trial conducted by Noopur et al. compared denosumab to zoledronic acid as a treatment for bone disease in newly diagnosed patients with MM. This study showed that denosumab was non-inferior to zoledronic acid in preventing skeletal-related events. Remarkably, denosumab demonstrated superior results in terms of the progression-free survival endpoint, providing further support for the efficacy of anti-RANKL therapy against myeloma. The reduction in risk of renal adverse events further supports the potential of denosumab as an additional treatment option for MM patients (180).

In OBs, their dysfunction is significantly influenced by the Dickkopf-1 (DKK1) produced by MM cells. Preclinical data demonstrated that BHQ880, a human DKK1 neutralizing antibody, could promote osteoblast differentiation while inhibiting the growth of myeloma cell and the formation of osteolytic lesions. These conclusions justify the need for clinical evaluation of BHQ880 in MM patients (181). While BHQ880 was well tolerated in a phase 1b trial showed that, its relative clinical activity could not be confirmed due to the accompanied administration of zoledronic acid and anti-myeloma therapy in the study (182).

### 4.6 BMSCs and vascular endothelial cells

Certain MM treatments aim to disrupt the interaction between MM cell and BMSCs and vascular endothelial cells. As previously mentioned, CXCR4 is essential for myeloma cell dissemination within and outside the BM. It acts as a pivotal regulator of EMD formation by inducing an EMT-like phenotype in MM, highlighting CXCR4 as a unique therapeutic target for patients with EMD and late-stage RRMM. Both *in vitro* and *in vivo* studies have shown that the downregulation of CXCR4 by therapeutic targeting or knockdown can impair the interaction between MM cells and BM milieu and increase their sensitivity to other therapeutic agents (183), providing a rationale for investigating its usage in clinical settings. Additionally, CXCR4 blockade decreases CD4+ T-cell exhaustion (184), enhances cytotoxic activity of immune cells (185), reverts the suppressive activity of Tregs (186), and modulates immunotherapy with anti-PD-1 (187), emphasizing its immunomodulatory effects. In a phase 1b/2 trial, ulocuplumab, a first-in-class fully human IgG4 monoclonal anti-CXCR4 antibody, demonstrated satisfactory safety and achieved an excellent overall response rate of 55.2% when combined with lenalidomide and dexamethasone in RRMM patients (188). This clinical trial supported CXCR4 inhibitors as an attractive class of anti-myeloma medications that deserve additional evaluation in larger clinical studies. Further research is required to explore the synergy between CXCR4 inhibitors and other anti-MM agents.

Also, the VEGF overexpression resulting from this interaction represents another potential target. Preclinical researches have shown that VEGF inhibition has activity against MM cells and synergizes with the proteasome inhibitor bortezomib (189, 190). However, in several clinical trials testing VEGF or VEGF receptor (VEGFR) inhibitors as single-agent treatment, none of them have shown significant clinical responses (191, 192). Similarly, combination regimens involving these inhibitors with IMiDs or with bortezomib have not yielded meaningful results (192, 193). Hence, future attempts to inhibit VEGF in MM patients ought to be made cautiously in patient selection and supported by a solid justification.

### 4.7 Others

Direct inhibition of VLA-4 has shown numerous beneficial effects in the MM microenvironment in preclinical studies. It can diminish MM stimulation by BM stroma, attenuate the pro-cancer signals initiated by MM-mesenchymal stromal cells (MSCs) microvesicles (MVs), abrogate angiogenesis induced by VEGF, block signaling pathways triggered by VEGF and IGF-1that promote MM cell migration, and restore sensitivity of MM cells treated with MM-MSCs MVs to doxorubicin and bortezomib (194, 195). According to these advantages, natalizumab, a recombinant humanized monoclonal antibody (mAb) against VLA-4 that is approved for Crohn’s disease and multiple sclerosis treatment, was evaluated in a phase 1/2 study (NCT00675428) for RRMM. Regrettably, this trial was prematurely terminated not due to safety concerns but due to insufficient patient enrollment. Therefore, alternative approaches are required to specifically target VLA-4 in the MM microenvironment.

Since hypoxic conditions have been identified in the BM of MM patients, evofosfamide, a 2-nitroimidazole prodrug of the DNA alkylator bromo-isophosphoramide (Br-IPM), has been specifically engineered to exhibit activity under low oxygen conditions, and demonstrated efficacy in both *in vitro* and *in vivo* preclinical models of MM (196). Evofosfamide also showed a synergistic effect when combined with bortezomib, leading to the promotion of apoptosis in MM cells (137). A phase 1/2 trial reveals that the administration of evofosfamide, with or without bortezomib, has excellent tolerability and results in stable disease and improved survival in patients with heavy prior treatments with advanced-stage RRMM (197). These data indicate that targeting the hypoxic microenvironment could be an effective new approach to the treatment of MM, emphasizing the need for further investigation.

## 5 Conclusion

The intricate interplay between the bone marrow (BM) microenvironment and myeloma cells plays a crucial role in supporting MM cell growth and facilitating immune evasion through diverse mechanisms. As a result, the tumor microenvironment is a promising therapeutic target, particularly for heavily pre-treated patients with end-stage RRMM. As such, multiple therapeutic strategies targeting different MM microenvironment components have been discovered and developed over the past decades to improve patient outcomes. Despite the significant advancements, most of the strategies failed to translate into clinical practice. Therefore, future research endeavors should focus on conducting larger-scale and multicenter studies to elucidate the mechanisms of action and resistance associated with different therapeutic approaches, identify new targets, and develop more effective, selective, and well-tolerated targeted therapies. This will contribute to improving patient outcomes and advancing the field of MM treatment.

## Author contributions

KL: Conceptualization, Writing – original draft. WW: Writing – review & editing, Data curation. YL: Writing – original draft. CX: Writing – review & editing. JL: Writing – review & editing. LX: Conceptualization, Writing – original draft, Writing – review & editing.
